# Short-course compared to long-course palliative radiotherapy for oesophageal cancer: a single centre observational cohort study

**DOI:** 10.1186/s13014-021-01880-9

**Published:** 2021-08-16

**Authors:** Halla Sif Ólafsdóttir, Fredrik Klevebro, Nelson Ndegwa, Gabriella Alexandersson von Döbeln

**Affiliations:** 1 Department of Clinical Science, Intervention and Technology, Karolinska Institutet, 141 52 Huddinge, Sweden; 2 Cancer Theme, Karolinska University Hospital, 171 64 Stockholm, Sweden; 3 Department of Medical Epidemiology and Biostatistics, Karolinska Institutet, 171 77 Stockholm, Sweden

**Keywords:** Oesophageal neoplasm, Palliative care, Radiotherapy, Dose fractionation, Dysphagia

## Abstract

**Background:**

Common symptoms of oesophageal cancer are dysphagia, pain, and bleeding. These symptoms can be relieved with palliative radiotherapy. The aim of this study was to analyse the outcome of two different palliative radiotherapy schedules.

**Methods:**

We conducted a retrospective cohort study on palliative radiotherapy for oesophageal cancer given at Karolinska University Hospital. Patients included were treated with either short-course (20 Gy in 4 Gy fractions daily, 5 consecutive workdays) or long-course (30–39 Gy in 3 Gy fractions, 10–13 consecutive workdays) palliative external beam radiotherapy between January 2009 and December 2013. The primary endpoint was dysphagia relief and secondary endpoints were adverse events, re-interventions, and overall survival. Cox regression analyses were used to estimate the effect of treatment schedule on survival.

**Results:**

A total of 128 patients received external beam radiotherapy under the study period, of these 75 (58.6%) received short-course radiotherapy and 53 (41.4%) long-course radiotherapy. Sixteen (30.8%) patients experienced dysphagia relief after short-course radiotherapy and 9 (22.0%) patients after long-course radiotherapy (*p* = 0.341). Acute toxicity was less frequent after short-course radiotherapy than after long-course radiotherapy, particularly oesophagitis (35.4% vs. 56.0%, *p* = 0.027) and nausea/emesis (18.5% vs. 36.0% *p* = 0.034). Re-interventions tended to be more common after short-course radiotherapy (32.0%) than after long-course radiotherapy (18.9%) (*p* = 0.098). There was no difference in overall survival between the two groups.

**Conclusions:**

Short- and long-course palliative radiotherapy for oesophageal cancer were equally effective to relieve dysphagia and no difference was seen in overall survival. Acute toxicity was, however, more frequent and more severe after long-course radiotherapy. Our results suggest that short-course radiotherapy is better tolerated with equal palliative effects as long-course radiotherapy.

## Background

A common presenting symptom of oesophageal cancer that significantly affects health-related quality of life is dysphagia [1]. Dysphagia can be alleviated and palliated by several different means such as local endoscopic measures with stenting, internal or external radiotherapy and/or chemotherapy [2]. Chemotherapy leads to partial or complete resolution of dysphagia in most patients [3–5]. Self-expanding metallic stents are effective for short-term relief from dysphagia, although not without complications [5–7]. Radiotherapy, either external beam radiotherapy (EBRT) or internal high dose-rate (HDR) brachytherapy, is an effective palliative treatment of dysphagia [8, 9] and has a more prolonged effect in comparison to stents [10]. Radiotherapy alone for dysphagia relief is as effective as concomitant palliative radio- and chemotherapy, however the latter comes at the cost of increased toxicity [11]. In summary, there are several ways to relieve malignant dysphagia, which one is preferred depends on tumour and patient characteristics, the discretion of the treating physician and availability of the different treatment modalities.

The prognosis of incurable oesophageal cancer is dismal, with a 5-year survival of 4—6% [12–14]. Overall survival of oesophageal cancer is marginally improved with palliative chemotherapy and targeted treatments compared to best supportive care, with a median overall survival of 4.7 months versus 4.2 months, respectively (HR 0.81; 95% CI 0.71–0.92) [15]. Considering the expected short survival time, palliative treatments should ideally be efficient and convenient with a minimum of side-effects. Different radiotherapy regimens are used according to local practice to palliate oesophageal cancer. The aim of this study was to analyse the effectiveness of two different palliative EBRT schedules: short-course radiotherapy; 4 Gy daily given 5 workdays in a row (SR) and long-course radiotherapy; 3 Gy daily for 10—13 consecutive workdays (LR), at a single institution, Karolinska University Hospital, which serves the whole Stockholm county.

## Methods

### Study design

We conducted a retrospective single-centre observational cohort study. Patients included had incurable oesophageal cancer or gastroesophageal junction cancer and were treated with palliative EBRT at Karolinska University Hospital between the 1st of January 2009 and the 31st of December 2013. The last date of follow-up was the 31st of December 2018. The study was approved by the Ethical Review Board in Stockholm (Diary Number 2018/724).

### Study cohort

**Fig. 1:**
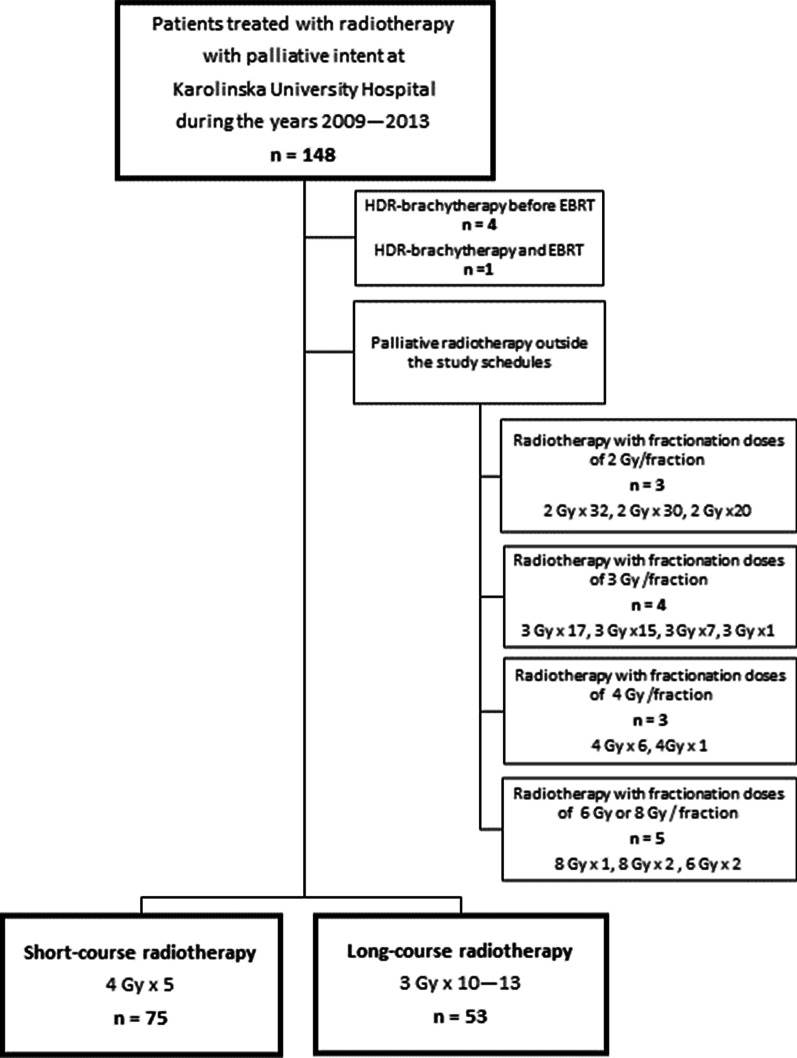
Study flow diagram of patient selection

Patients were identified in the hospital’s electronic charts by ICD-10 codes (C15.0, C15.3-C15.5, C15.8, C15.9 and C16) and palliative intent of radiotherapy. Palliative intent was defined as patients that had primary metastatic disease, relapsed after initial treatment with curative intent, or were assessed unfit for surgery or curatively intended oncological treatment. In total, 148 patients received radiotherapy with palliative intent under the study period. Patients treated with EBRT outside the SR or LR dose schedules (n = 15) were excluded, as well as patients treated with HDR-brachytherapy before the EBRT or HDR-brachytherapy in the same session as the EBRT (n = 5). See Fig. 1 for the study flow diagram. The EBRT planning was done on a preparation computed tomography scan. The radiotherapy was delivered with photons of either 18 MV or 6 MV energy. Three-dimensional conformal radiotherapy technique, with either two or four fields (box technique), was used. At the time, volumetric modulated arc therapy and intensity-modulated radiotherapy was not used at our department for patients treated with palliative intent.

### Study outcomes

The primary outcome of the study was improvement of dysphagia by at least one grade according to the Common Terminology Criteria for Adverse Events, version 5.0 (CTCAE v5.0) [16]. Secondary outcomes were treatment related toxicity, other adverse events of grade ≥ 3, re-interventions after the radiotherapy and overall survival (OS).

### Data collection

Medical files from the hospital’s electronic charts and radiotherapy planning system were screened by the first author. If there was ambiguity regarding any variable a senior clinical oncologist was consulted. Baseline characteristics collected included age, sex, WHO performance status, body mass index (BMI kg/m^2^), enteral tube feeding, indications for radiotherapy, comorbidity registered in medical charts at the time or before the given radiotherapy and scored according to the Charlson Comorbidity Index (CCI) [17] (omitting age and cancer in the calculation of the score), tumour stage and location according to the American Joint Committee on Cancer (7th edition of TNM staging system) [18] and tumour histology.

The charts were screened for descriptions of dysphagia at the last appointment before the radiotherapy and at the first follow-up after the radiotherapy. Acute toxicity was defined as adverse events related to the radiotherapy, occurring within six weeks from the start of the radiotherapy. All other adverse events of grade ≥ 3 with uncertain relation to the radiotherapy, occurring from the start of radiotherapy until the last date of follow-up, were classified separately. Dysphagia and toxicities were graded according to CTCAE v5.0 [16]. Details of palliative chemotherapy (before, during or after the radiotherapy) and re-interventions (repeat EBRT, HDR-brachytherapy or stent placement) were also recorded. Re-interventions were defined as interventions applied after the first EBRT until death or last date of follow-up, whichever came first. OS was defined as the time from the start of radiotherapy until death or the last date of follow-up. Date of death was collected from the patients’ medical charts.

### Statistical analysis

Patient and tumour characteristics were presented with descriptive statistics. *χ*^2^ or Fisher’s exact test were used to analyse differences in categorical variables and Wilcoxon Rank Sum test for differences in continuous variables. Dysphagia grade, acute toxicity of all grades, other adverse events of grade ≥ 3 and re-interventions were also compared with *χ*^2^ or Fisher’s exact test. Survival time was estimated with the Kaplan–Meier method and the log-rank test used to assess differences in overall survival of the two treatment schedules. Living patients were censored at the last date of follow-up. Univariable and multivariable Cox regression analyses were applied to determine predictors of overall survival. Covariates assessed with the univariable model were age (≤ 70 vs. > 70 years), sex (male vs female), histology (adenocarcinoma vs squamous cell carcinoma), location (proximal/middle vs distal), T stage (T0–3 vs. T4), N stage (N0 vs N +), M stage (M0 vs M1), CCI score (0 vs. ≥ 1), cancer presentation (primary vs recurrent disease), BMI (≥ 20 vs. < 20 kg/m^2^) and radiotherapy schedule. Covariates assessed with the multivariable model were age (≤ 70 vs. > 70 years), sex (male vs female), histology (adenocarcinoma vs squamous cell carcinoma), tumour location (proximal/middle vs distal), M stage (M0 vs M1) and CCI score (0 vs. ≥ 1). An assessment of the proportional hazards assumption was performed for all models using the Grambsch and Therneau test based on Schoenfeld residuals [19]. The results of the analyses were reported as hazard ratios (HR), with 95% confidence intervals (CI). A *p* value of < 0.05 was regarded as statistically significant. Stata (v16.0) was used for statistical data analyses.

## Results

Between 1st of January 2009 and 31st of December 2013, 128 patients received palliative radiotherapy for oesophageal cancer with either SR or LR at Karolinska University Hospital. Of these, 75 (58.6%) patients were prescribed SR and 53 (41.4%) patients LR. Three of the patients that were prescribed 20 Gy received 16 Gy in four fractions. One patient received 20 Gy in four 5 Gy fractions. These four patients were all included in the SR group.

### Patient characteristics

The median age was 71 years in the whole group. Most of the patients were male (78.9%), had an adenocarcinoma (AC) (57.8%) and a distal location of the primary tumour (68.8%). Metastatic disease was more common in the SR group (69.3%) than in the LR group (39.6%) (*p* < 0.001). Patients treated with SR had higher dysphagia grade at baseline compared to patients treated with LR (*p* = 0.022). The difference between the two treatment groups in other baseline characteristics was not statistically significant. However, a tendency was seen towards older age, increased frequency of squamous cell carcinoma (SCC) and a lower T-stage in the LR group. Details of baseline characteristics are presented in Table 1.

**Table 1:** Baseline characteristics of patients treated with palliative radiotherapy for oesophageal cancer

	All patients n (%)	Short-course radiotherapy 4 Gy × 5 n (%)	Long-course radiotherapy 3 Gy × 10–13 n (%)	*p* value
All patients	128	75 (58.6)	53 (41.4)	
Age, years, median	71 (IQR 64–80)	69 (IQR 63–78)	73 (IQR 67–81)	0.082
**Sex**				0.215
Male	101 (78.9)	62 (82.7)	39 (73.6)	
Female	27 (21.1)	13 (17.3)	14 (26.4)	
**Histology**				0.087
Adenocarcinoma	74 (57.8)	47 (62.7)	27 (50.9)	
Squamous cell carcinoma	50 (39.1)	24 (32.0)	26 (49.1)	
Unspecified*	4 (3.1)	4 (5.3)	0 (0)	
**Location of primary tumour**				0.547
Proximal	19 (14.8)	10 (13.3)	9 (17.0)	
Middle	17 (13.3)	8 (10.7)	9 (17.0	
Distal	88 (68.8)	53 (70.7)	35 (66.0)	
Unspecified*	4 (3.1)	4 (5.3)	0 (0)	
**T stage**				0.061
T1	2 (1.6)	0 (0.0)	2 (3.8)	
T2	6 (4.7)	1 (1.3)	5 (9.4)	
T3	35 (27.3)	19 (25.3)	16 (30.2)	
T4	24 (18.8)	16 (21.3)	8 (15.1)	
Unknown*	61 (47.7)	39 (52.0)	22 (41.5)	
**N stage**				0.153
N0	9 (7.0)	3 (4.0)	6 (11.3)	
N +	89 (69.5)	55 (73.3)	34 (64.2)	
Unknown*	30 (23.4)	17 (22.7)	13 (24.5)	
**M stage**				< 0.001
M0	49 (38.3)	19 (25.3)	30 (56.6)	
M1	73 (57.0)	52 (69.3)	21 (39.6)	
Unknown*	6 (4.7)	4 (5.3)	2 (3.8)	
**Cancer presentation**				0.447
Primary disease	121 (94.5)	72 (96.0)	49 (92.5)	
Recurrent disease	7 (5.5)	3 (4.0)	4 (7.6)	
Indications for radiotherapy**				
Dysphagia	112 (87.5)	69 (92.0)	43 (81.1)	
Tumour haemorrhage**	27 (21.1)	19 (25.3)	8 (15.1)	
Weight loss	57 (44.5)	35 (46.7)	22 (41.5)	
Pain	28 (21.9)	18 (24.0)	10 (18.9)	
Other	17 (13.3)	9 (12.0)	8 (15.1)	
**Dysphagia grade according to CTCAE v5.0**				0.022
0	17 (13.6)	6 (8.2)	11 (21.2)	
1	18 (14.4)	7 (9.6)	11 (21.2)	
2	47 (37.6)	33 (45.2)	14 (26.9)	
3	43 (34.4)	27 (37.0)	16 (30.8)	
**Tube feeding**				0.740
Yes	39 (30.5)	22 (29.3)	17 (32.1)	
No	89 (69.5)	53 (70.7)	36 (67.9)	
**Body mass index (kg/m**^**2**^**)**				0.191
< 20	27 (21.1)	14 (18.7)	13 (24.5)	
20—24.9	42 (32.8)	29 (38.7)	13 (24.5)	
≥ 25	30 (23.4)	15 (20.0)	15 (28.3)	
Unknown*	29 (22.7)	17 (22.7)	12 (22.6)	
**Charlson Comorbidity Index score**				0.323
0	57 (44.5)	36 (48.0)	21 (39.6)	
≥ 1	68 (53.1)	37 (49.3)	31 (58.5)	
Unknown*	3 (2.3)	2 (2.7)	1 (1.9)	
**Oesophageal stent**	40 (31.3)	22 (29.3)	18 (34.0)	0.578
**Chemotherapy before the EBRT**	25 (19.5)	17 (22.7)	8 (15.1)	0.287

^*^Category not included in the *p* value calculations

^**^No calculation of *p* as the same patient could have more than one indication for radiotherapy

^***^Tumour haemorrhage = Haematemesis, melena or anaemia caused by primary tumour

CTCAE v5.0 = Common Terminology Criteria for Adverse Events, version 5.0

IQR = interquartile range

### Dysphagia

There was no difference in dysphagia relief between the two treatment groups. The median time from radiotherapy to follow-up was five weeks (interquartile range (IQR) 3–8 weeks). Information on dysphagia grade was missing at baseline in 3 (2.3%) patients in the whole group and at follow-up in 23 (30.7%) patients after SR and in 12 (22.6%) patients after LR (*p* = 0.316). Sixteen (30.8%) patients had improvement of dysphagia by at least one grade after SR, and 9 (22.0%) patients after LR (*p* = 0.341), according to CTCAE v.5.0. Eight (15.4%) patients in the SR group and 9 (22.0%) patients in the LR group experienced worsening of dysphagia by at least one grade (*p* = 0.416). After omitting the 17 patients who had no dysphagia at baseline from the calculations there was still no difference (*p* = 0.365) in the effect on dysphagia between the two treatment schedules.

### Toxicity and adverse events

Acute toxicity from the radiotherapy (defined as related adverse events that occurred within six weeks from the start of the radiotherapy treatment) is summarized in Table 2. Grade ≥ 3 acute toxicity was less common after SR than after LR; two (3.1%) patients experienced acute toxicity of grade ≥ 3 after SR and 8 (16.0%) patients after LR (*p* = 0.020). No acute toxicity was seen in 21 (32.3%) patients after SR and in six (12.0%) patients after LR (*p* = 0.011). One patient was treated for grade 4 radiation induced pneumonitis, eight weeks after LR.

**Table 2:** Acute toxicity within six weeks from palliative radiotherapy for oesophageal cancer

	All patients n (%)	Short-course radiotherapy 4 Gy × 5 n (%)	Long-course radiotherapy 3 Gy × 10–13 n (%)	*p* value
Oesophagitis	51 (44.4)	23 (35.4)	28 (56.0)	0.027
Oesophagitis grade ≥ 3	7 (6.1)	1 (1.5)	6 (12.0)	0.042
Nausea/Emesis	30 (26.1)	12 (18.5)	18 (36.0)	0.034
Nausea/Emesis grade ≥ 3	1 (0.9)	1 (1.5)	0 (0.0)	1.000
Fatigue	38 (33.0)	19 (29.2)	19 (38.0)	0.322
Fatigue grade ≥ 3	2 (1.7)	0 (0.0)	2 (4.0)	0.187
Oesophageal pain requiring opiates	23(21.1)	10 (16.4)	13 (27.1)	0.175
Other*	19 (16.5)	10 (15.4)	9 (18.0)	0.708

^*^Fever, pain, cough, hoarseness, diarrhoea, constipation, and loss of appetite

Other adverse events of grade ≥ 3 (defined as adverse events that occurred under the whole study period and of uncertain relation to the radiotherapy) occurred in 27 (36.0%) patients after SR and in 24 (45.3%) patients after LR (*p* = 0.291), for details see Table 3. Fatal (grade 5) adverse events occurred in 5 (6.7%) patients after SR (3 tumour haemorrhages and 2 oesophageal perforations) and in 6 (11.3%) patients after LR (4 tumour haemorrhages, 1 oesophageal perforation and 1 tracheal stenosis) (*p* = 0.524). All the fatal events were considered related to local tumour burden.

**Table 3:** Adverse events of grade ≥ 3 after palliative radiotherapy for oesophageal cancer

	All patients n (%)	Short-course radiotherapy 4 Gy × 5 n (%)	Long-course radiotherapy 3 Gy × 10–13 n (%)	*p* value
Oesophageal perforation	7 (5.5)	6 (8.0)	1 (1.9)	0.238
Oesophageal stenosis/obstruction	21 (16.4)	13 (17.3)	8 (15.1)	0.736
Tumour haemorrhage*	23 (18.0)	11 (14.7)	12 (22.6)	0.247
Tracheal fistulae	1 (0.8)	0 (0.0)	1 (1.9)	1.000
Tracheal stenosis	3 (2.3)	1 (1.3)	2 (3.8)	0.569
Aspiration pneumonia	2 (1.6)	1 (1.3)	1 (1.9)	1.000
Oesophageal pain	2 (1.6)	1 (1.3)	1 (1.9)	1.000

^*^Tumour haemorrhage = Haematemesis, melena or anaemia caused by the primary tumour

### Re-interventions

Twenty-four (32.0%) patients underwent re-interventions after SR and 10 (18.9%) patients after LR (*p* = 0.098). Seven (9.3%) patients were treated with re-irradiation after SR (5 patients EBRT and 2 patients HDR-brachytherapy) and 1 (1.9%) patient after LR (HDR-brachytherapy) (*p* = 0.139). A stent was placed in the oesophagus in 20 patients (26.7%) after SR and in 9 (17.0%) patients after LR (*p* = 0.197). The median length of time from radiotherapy to re-intervention was 122 days after SR and 119 days after LR. None of the patients were lost to follow-up.

### Survival

**Fig. 2:**
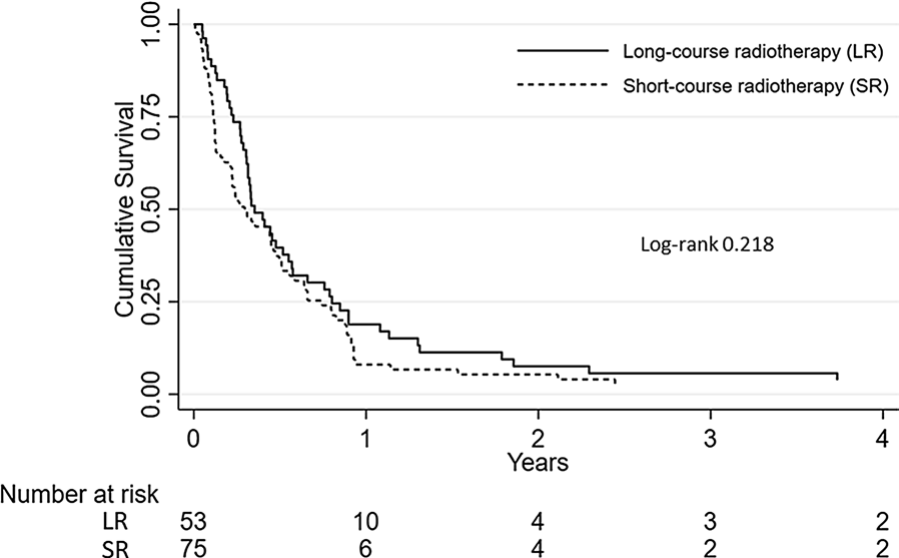
Kaplan–Meier estimates of overall survival for short-course and long-course palliative radiotherapy for oesophageal cancer

In the whole group the median OS was 122 days (IQR 47–290); one-year OS was 12.5% (95% CI, 7.5—18.9%) and two-year OS was 6.3% (95% CI, 2.9–11.3%). The median OS after SR was 108 days (IQR 41–272) and 129 days (IQR 84–293) after LR (*p* = 0.218), HR 1.25 (95% CI 0.87–1.79, *p* = 0.220). Survival curves for the two treatment schedules are displayed in Fig. 2. At the last date of follow-up three patients were still alive, all three patients had survived at least five years. Two of these patients had AC and were treated with SR, the third patient had SCC and was treated with LR.

**Fig. 3:**
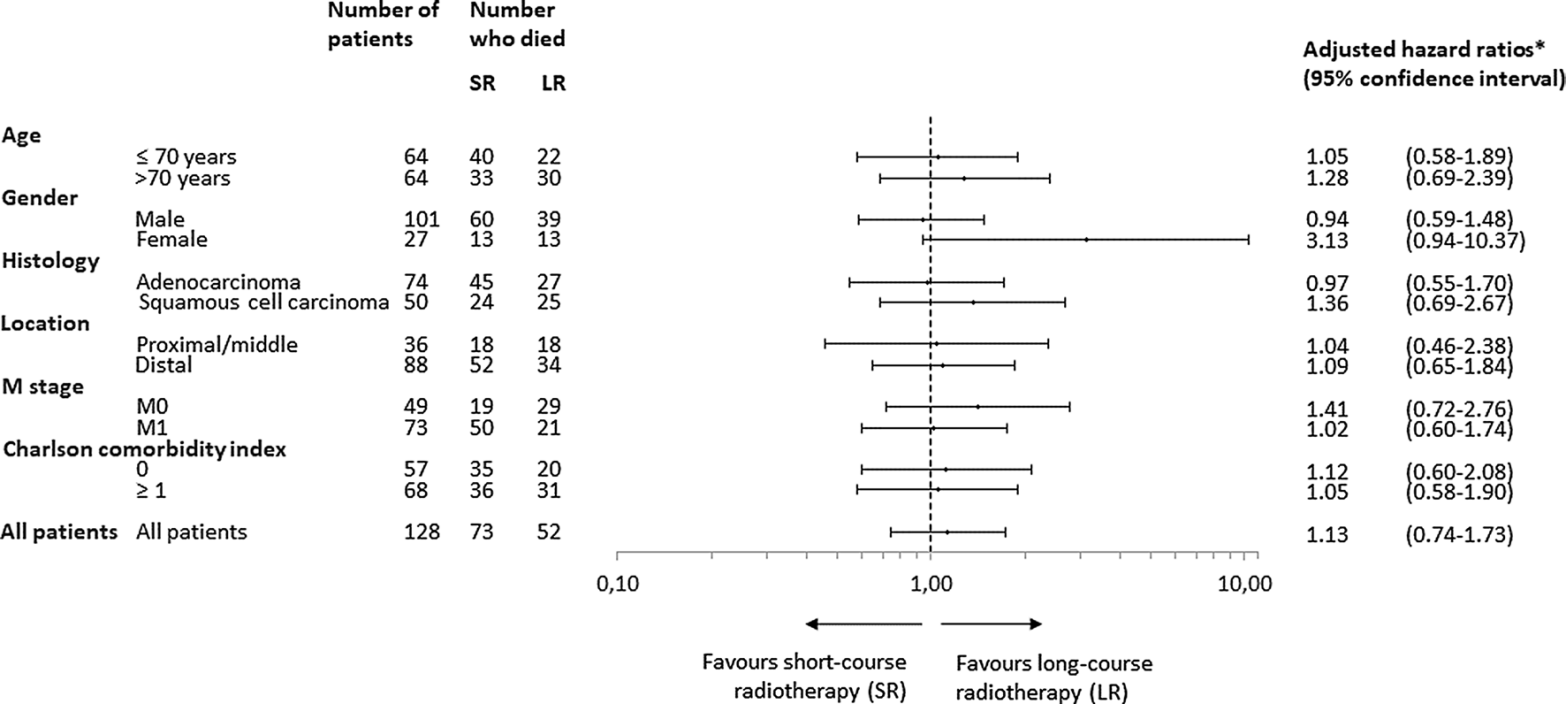
Adjusted hazard ratios of survival within subgroups of oesophageal cancer patients treated with palliative radiotherapy. *Hazard ratios were adjusted for age, sex, histology, tumour location, M stage and CCI score

None of the baseline characteristics had a statistically significant association with survival in univariable survival analyses (age, sex, histology, location, T stage, N stage, M stage, CCI score, cancer presentation, and BMI). In a multivariable survival analysis (age, sex, histology, tumour location, M stage and CCI score) distant metastases was the single variable with a statistically significant association with survival (HR 1.70, 95% CI 1.05–2.74). After fitting Cox models, an assessment of violation of the proportional hazards assumption showed no evidence for non-proportional effects (global test *p* = 0.8436). To assess if certain patient groups benefited from either of the two radiotherapy schedules a Cox regression analysis, adjusted for baseline variables (age, sex, histology, tumour location, tumour stage and CCI score), was used and HR calculated for each patient group. The results are presented in Fig. 3. Neither of the two treatment schedules was shown to lead to a survival advantage for any of the subgroups, though a tendency was seen towards a benefit from LR compared to SR in women, patients older than 70 years, tumours of squamous cell histology and patients without distant metastases.

## Discussion

Our results demonstrate equal effectiveness of SR and LR for short-term relief of dysphagia caused by oesophageal cancer. No difference was seen in survival. However, acute toxicity was more frequent and more severe after LR than after SR. These results emphasize the importance of careful consideration when choosing a radiotherapy schedule. Longer radiotherapy schedules, in a patient group with dismal prognosis, might lead to more side-effects without greater symptom relief or better survival.

Previously, radiotherapy has been shown to relieve dysphagia in 41—75% of patients [9, 20–23]. No statistically significant difference was seen between SR and LR in dysphagia relief in our study. Vermeulen et al. [20] did a cohort study in the Netherlands on low-dose radiotherapy (4 Gy × 5 EBRT) compared to high-dose radiotherapy (3 Gy × 10 EBRT and 12 Gy single dose HDR-brachytherapy). Dysphagia improvement was reported in 50% of patients treated with low-dose radiotherapy and in 66% of patients treated with high-dose radiotherapy, the difference was not statistically significant. In another Dutch study, Walterbos et al. [21] did a retrospective cohort study on three different EBRT schedules: 4 Gy × 5, 3 Gy × 10 and 3 Gy × 13. In this study, the dysphagia score improved in 41% of patients and no difference was found in dysphagia relief between the three schedules. Thus, neither our nor the Dutch results suggest that higher radiotherapy doses are more likely to lead to short-term relief from malignant dysphagia than lower radiotherapy doses.

Short-term symptom relief from radiotherapy can be hampered by acute side-effects, although previous studies have reported acute toxicities of grade ≥ 3 from palliative radiotherapy as uncommon (3–3.9%) [21–23]. In our cohort, 9% of the patients experienced grade ≥ 3 acute toxicities and this was less frequent after SR than after LR. Vermeulen et al. [20] reported severe adverse events in 15% of the patients and no difference between low-dose (4 Gy × 5 EBRT) and high-dose radiotherapy (3 Gy × 10 EBRT and 12 Gy single dose HDR-brachytherapy). We chose to separate acute toxicity from other adverse events, which could possibly explain the differences between our results and Vermeulen’s. In our study, acute toxicity had a clear causal relationship to the treatment and occurred within 6 weeks. Other adverse events had a possible and perhaps a more probable relation to locoregional tumour burden. Like Vermeulen’s results, we saw no statistically significant differences between the two studied treatment schedules in other adverse events of grade ≥ 3. Considering the poor prognosis of incurable oesophageal cancer and the more moderate acute toxicity profile of SR compared to LR presented in our study, SR might be the preferred treatment schedule.

Though the prognosis is generally dismal for incurable oesophageal cancer, a few patients live long enough for the short-term symptom relief to subside and some require re-interventions. Ideally, the length of dysphagia improvement should be recorded with a follow-up dysphagia score. However, the short survival of the patients limited the available data on the length of dysphagia relief. On the other hand, re-intervention reflects an important part of the outcome of the treatment, both for patients and health care providers. Walterbos’ [21] study on three different EBRT schedules found that 24% of patients underwent re-intervention in the group as a whole; 35% of patients after radiotherapy with 4 Gy × 5, 22% of patients after radiotherapy with 3 Gy × 10 and 17% of patients after radiotherapy with 3 Gy × 13. Furthermore, treatment schedule was the only prognostic factor related to time to re-intervention. Similarly, Vermeulen et al. [20] found that re-treatment was indicated in 23% of patients after low-dose radiotherapy (4 Gy × 5) and 17% of patients after high-dose radiotherapy (3 Gy × 10 and 12-Gy single-dose HDR-brachytherapy). In our cohort, there was a greater need for a re-intervention after SR than after LR (32% vs 19%), though the difference was not statistically significant. The median time from radiotherapy to re-intervention in our study was similar in the two groups, 122 days after SR and 119 after LR. Altogether, these three retrospective studies indicate that the need for re-intervention after radiotherapy with lower doses is greater than after radiotherapy with higher doses. Whether this outweighs the longer treatment times with increased risk of toxicity remains to be established.

Increased temporary acute toxicity might be acceptable if a lesser need for re-intervention and longer survival were to be expected. In our study, survival was similar after treatment with higher and lower radiotherapy doses. However, other observational studies have shown that higher radiotherapy doses in palliative settings might improve survival for patients with oesophageal cancer. Guttman et al. [24] did a cohort study on radio- and chemotherapy in patients with metastatic oesophageal cancer based on the National Cancer Database in the USA. Their results demonstrated better OS after higher doses (≥ 50.4 Gy) of radiotherapy compared to lower doses (< 50,4 Gy), median overall survival was 11.3 months and 7.5 months, respectively. Vermeulen et al. [20] reported better survival in patients treated with EBRT of 3 Gy × 10 and 12-Gy single-dose HDR-brachytherapy compared to patients treated with EBRT of 4 Gy × 5, median survival was 177 days versus 88 days. Lastly, survival after EBRT with 3 Gy × 13 was superior to survival after 4 Gy × 5 in the study of Walterbos et al., where median OS was 9.7 months versus 4.6 months [21]. A possible explanation of superior survival after longer radiotherapy schedules in retrospective studies could be a selection bias. In the study by Walterbos et al. [21] patients selected for the different treatment schedules differed in baseline characteristics. For example, patients with distant metastases were more often treated with lower radiotherapy doses, which was associated with worse survival in their study as well as in ours. Vermeulen et al. [20] and Guttmann et al. [24] used propensity score to account for confounding, however residual confounding cannot be adjusted for.

Nevertheless, there seem to be groups of patients with more favourable prognosis who might well benefit from higher radiation doses and longer therapies. Women with localised SCC and ≥ 55 years of age were demonstrated to have better survival in two cohort studies, one based on data from the Surveillance, Epidemiology and End Results Program in the USA [25] and the other a nationwide Swedish cohort study [26] on the prognosis of patients treated with surgery for oesophageal cancer. Similarly, female sex was shown to be a positive prognostic survival factor in a Chinese study on the impact of sex on the prognosis of SCC in the oesophagus treated with definitive radiotherapy [27]. In our subgroup analysis, we saw a tendency towards better survival for women, older patients (> 70 years), SCC and patients with localised disease. These patient groups might benefit from longer radiotherapy treatments, though further research is needed before applying this in clinical practice.

Our study has some limitations that should be addressed. Firstly, the inherent selection bias of a retrospective study. The baseline characteristics of the two radiotherapy groups differed, although the only variable that differed significantly was distant metastases. To minimize bias and improve internal validity a randomized controlled trial would be ideal. Still, to our knowledge, a randomized controlled trial on different palliative EBRT schedules for oesophageal cancer has not been done and the results from our study contribute to the evidence on which we can base current treatment decisions. Secondly, the retrospective nature of the study led to limitations in registered data and missing values. Missing data would have been minimized by prospectively collected standardised data. Though, the consistency of data was strengthened by the availability of all medical charts, no loss to follow-up and because all medical charts were screened by one person, the first author. Thirdly, the precision of the results would have improved with a larger study population, though the real-life nature of the study, demonstrating clinical practice in a high-volume tertiary cancer centre, strengthens the study’s generalisability.

## Conclusions

Longer radiotherapy schedules were not associated with better resolution of dysphagia or longer survival than shorter schedules in patients treated with palliative intent for oesophageal cancer. After treatment with longer radiotherapy schedules acute toxicity was more frequent and more severe. The generally short survival of patients with incurable oesophageal cancer highlights the importance of choosing effective treatment with minimum toxicity. In this context, we conclude that short-course radiotherapy is a valid palliative radiotherapy treatment although there might be certain patient groups that could benefit from longer radiotherapy schedules.

## Data Availability

The datasets generated and analysed during the current study are not publicly available as individual privacy could be compromised but are available from the corresponding author on reasonable request.

## Abbreviations

AC: Adenocarcinoma
CCI: Charlson Comorbidity Index
EBRT: External beam radiotherapy
IQR: Interquartile range
HDR: High dose-rate
LR: Long-course radiotherapy
OS: Overall survival
SCC: Squamous cell carcinoma
SR: Short-course radiotherapy
